# The Interaction Between PGD2 and G6PD6 Is Involved in Aromatic Amino Acid Synthesis

**DOI:** 10.3390/biology14121712

**Published:** 2025-11-30

**Authors:** Qian Tang, Zhuanglin Shen, Jiaqin Huang, Dingxuan Zhang, Qiao Zhao

**Affiliations:** 1 School of Applied Biology, Shenzhen City Polytechnic, Shenzhen 518116, China; 2 CAS Key Laboratory for Quantitative Engineering Biology, Shenzhen Institute of Synthetic Biology, Shenzhen Institute of Advanced Technology, Chinese Academy of Sciences, Shenzhen 518055, China; 3 Shenzhen Key Laboratory of Synthetic Genomics, Guangdong Provincial Key Laboratory of Synthetic Genomics, Key Laboratory of Quantitative Synthetic Biology, Shenzhen Institute of Synthetic Biology, Shenzhen Institute of Advanced Technology, Chinese Academy of Sciences, Shenzhen 518055, China

**Keywords:** PGD2, G6PD6, aromatic amino acids, protein–protein interaction, *Arabidopsis thaliana*, oxidative pentose phosphate pathway

## Abstract

The biosynthesis of AAAs in plants primarily relies on the shikimate pathway, with metabolic flux sustained by NADPH and E4P generated via the OPP pathway. However, how OPP enzymes coordinate to support AAA production remains unclear. Here, we investigated the direct interaction between two consecutive NADPH-producing enzymes, G6PD6 and PGD2, and its role in metabolic coupling. Using BiFC, Co-IP, pull-down assays, and domain mapping, we showed that G6PD6 and PGD2 form a cytosolic protein complex via the C-terminal domain of PGD2. Structural modeling identified potential interaction residues: PHE^294^, GLY^297^, and LEU^298^ in PGD2, and GLY^351^, LYS^499^, and ALA^500^ in G6PD6. Overexpression of either enzyme partially rescued the dwarf phenotype of *adh2* mutants caused by AAA deficiency. These findings indicate that the PGD2–G6PD6 complex coordinates OPP-derived reductive power and carbon flux to support downstream AAA biosynthesis. This study reveals a functional link between OPP enzyme interactions and AAA production, suggesting that metabolic flux can be regulated through direct enzyme–enzyme association. Future work will explore how this complex responds to metabolic demand and whether additional components contribute to coordinating flux between the OPP and shikimate pathways.

## Simple Summary

Plants rely on aromatic amino acids (AAAs) for the synthesis of proteins and secondary metabolites such as flavonoids and auxins. The oxidative pentose phosphate (OPP) pathway provides the shikimate pathway with the key precursor erythrose-4-phosphate (E4P) and the reducing equivalent NADPH, but the regulatory association between these two pathways has rarely been reported. This study confirmed that glucose-6-phosphate dehydrogenase 6 (G6PD6) and 6-phosphogluconate dehydrogenase 2 (PGD2), the rate-limiting enzymes in the OPP pathway, exhibit a co-expression pattern and interact directly; overexpression of either gene in the AAA synthesis-deficient *arogenate dehydrogenase 2* (*adh2*) mutant partially rescued the mutant’s nutritional deficiency phenotype. This finding clarifies the molecular mechanism by which G6PD6 and PGD2 synergistically participate in AAA synthesis and provides an important experimental basis for deciphering the regulatory network of plant AAA metabolism.

## 1. Introduction

Aromatic amino acids (AAAs) serve not only as essential building blocks for protein biosynthesis but also as key precursors for numerous secondary metabolites, including lignin, flavonoids, and auxins [1,2]. In plants, AAA biosynthesis depends on the shikimate pathway, which requires a steady supply of erythrose-4-phosphate (E4P) and NADPH from the oxidative pentose phosphate (OPP) pathway to sustain carbon flux and redox balance [3]. Notably, while the shikimate pathway primarily operates in plastids, the cytoplasmic OPP pathway supports AAA biosynthesis by transferring NADPH into plastids via metabolic shuttles [4] and E4P through phosphate pentose transporters (PPTs) [5], with a cytoplasmic AAA biosynthetic pathway also contributing to this process [6]. As the rate-limiting enzyme of the OPP pathway, glucose-6-phosphate dehydrogenase 6 (G6PD6) undergoes phosphorylation [7], persulfidation [8], and oligomerization [8], modifications that enhance its activity and consequently increase NADPH and carbon skeleton production [9]. 6-Phosphogluconate dehydrogenase 2 (PGD2), another major NADPH-producing enzyme in the OPP pathway, is the only isoform exhibiting peroxisomal and cytosolic dual subcellular localization and is essential for viability, underscoring its importance in plant metabolism [10,11].

Recent phenotypic complementation screens using the AAA-deficient *adh2* mutant [12] have identified several regulators of AAA metabolism. These include site-specific mutations in 3-dehydroquinate synthase (DHS) that reduce product inhibition and thereby increase pathway flux [13], as well as the aminotransferase REVERSAL OF SAV3 PHENOTYPE 1 (VAS1), which synthesizes phenylalanine, tyrosine, and tryptophan in the cytosol to fine-tune AAA levels [6]. Within this research framework, we previously found that a point mutation in PGD2 partially rescued the AAA deficiency of the *adh2* mutant by enhancing PGD2’s catalytic efficiency and metabolite turnover [14]. This observation suggested that PGD2 may function within a protein complex to modulate AAA biosynthesis through coordinated regulation of OPP pathway activity.

Co-expression analyses have shown that G6PD6 and PGD2 display highly synchronized expression patterns, yet whether these enzymes interact functionally and how such interaction may influence AAA synthesis has remained unresolved. Genetic evidence further indicates that overexpression of either enzyme partially alleviates the growth defects of AAA-deficient mutants, hinting at a potential synergistic role in controlling OPP pathway flux.

Here, we showed that PGD2 directly interacts with G6PD6 in the cytoplasm through the C-terminal domain of PGD2, forming a functional enzyme–enzyme complex. This interaction was supported by bimolecular fluorescence complementation, co-immunoprecipitation, pull-down assays, domain mapping, and structural modeling. Moreover, overexpression of either PGD2 or G6PD6 mitigated the growth defects of the *adh2* mutant, highlighting the physiological relevance of this complex. Together, our results reveal an uncharacterized regulatory connection between the OPP pathway and AAA biosynthesis and provide a mechanistic basis for how enzymes within the OPP pathway cooperate to coordinate metabolic flux.

## 2. Materials and Methods

### 2.1. Plant Materials and Growth Conditions

*Arabidopsis thaliana* wild-type (Columbia-0, Col-0) seeds were obtained from the Arabidopsis Biological Resource Center (ABRC, Carlsbad, CA, USA). The *adh2* mutant (SALK_001765, Col-0 background) was kindly provided by Dr. Qiao Zhao (Chinese Academy of Sciences, Shenzhen, China) [6].

Seeds were surface-sterilized in 75% (*v*/*v*) ethanol for 1 min and plated on half-strength Murashige and Skoog (1/2 MS) medium (Caisson Labs, Smithfield, UT, USA) containing 1% (*w*/*v*) sucrose and 0.6% (*w*/*v*) agar. After stratification at 4 °C in darkness for 3 days, seedlings were grown under long-day conditions (16 h light/8 h dark) at 22 °C with a light intensity of 120 µmol m^−2^ s^−1^. Two-week-old seedlings were transferred to a soil–vermiculite mixture (1:1, *v*/*v*) and maintained under the same growth conditions for subsequent phenotypic observations.

### 2.2. Plasmid Construction and Plant Transformation

The full-length coding sequences (CDSs) of *PGD2* (*At3g02360*) and *G6PD6* (*At5g40760*) were amplified and cloned into a modified pCAMBIA1300 vector containing the CaMV 35S promoter and a C-terminal YFP tag, generating the constructs 35S::PGD2-YFP and 35S::G6PD6-YFP. For co-immunoprecipitation (Co-IP) assays, PGD2 was cloned into pCAMBIA1300 with a C-terminal HA tag (35S::PGD2-HA), and G6PD6 was cloned into pCAMBIA1300 with a C-terminal GFP tag (35S::G6PD6-GFP). The *β-glucuronidase* gene (*GUS*) was inserted into the same GFP vector to generate a negative control (35S::GUS-GFP).

All plasmids were introduced into *Agrobacterium tumefaciens* strain GV3101 using the freeze–thaw method. Arabidopsis transformation was performed using the floral-dip procedure [15]. Transgenic seedlings were selected on 1/2 MS medium supplemented with 40 μg mL^−1^ hygromycin B (Sigma-Aldrich, St. Louis, MO, USA).

### 2.3. Subcellular Localization and Bimolecular Fluorescence Complementation (BiFC) Assay

To examine the subcellular localization of PGD2, truncated PGD2 (sPGD2; lacking the N-terminal 15 amino acids), and G6PD6, YFP fusion constructs were generated using different tagging strategies. sPGD2 was fused to YFP at its N-terminus (35S::YFP-sPGD2), whereas full-length PGD2 and G6PD6 were fused to YFP at their C-terminus (constructs 35S::PGD2-YFP and 35S::G6PD6-YFP, respectively). All constructs were introduced into *Agrobacterium tumefaciens* strain GV3101 and transiently expressed in leaves of 4-week-old *Nicotiana benthamiana* plants via *Agrobacterium* infiltration (OD_600_ = 0.5, containing 100 μM acetosyringone). Fluorescence signals were detected 3 days after infiltration using a confocal laser scanning microscope (Nikon, Melville, NY, USA).

For the BiFC assay, the full-length coding sequences of the indicated genes were cloned into the pCNHP-CYFP and pCNHP-NYFP vectors to generate C-terminal or N-terminal YFP-split fusions [16]. The constructs were transformed into GV3101 and infiltrated into *N. benthamiana* leaves under the same conditions as above. YFP fluorescence was excited at 488 nm and detected at 500–530 nm. mCherry signals were monitored at 543 nm excitation and 580–620 nm emission. Chlorophyll autofluorescence was visualized using 635 nm excitation and 650–750 nm emission.

### 2.4. Co-Immunoprecipitation (Co-IP) Assay

For in vivo verification of the PGD2–G6PD6 interaction, *Agrobacterium tumefaciens* strains carrying 35S::PGD2-HA and 35S::G6PD6-GFP (or 35S::GUS-GFP as a negative control) were mixed at a 1:1 OD_600_ ratio, supplemented with 100 μM acetosyringone, and incubated at 28 °C for 1 h. The mixture was then infiltrated into the leaves of 4-week-old *Nicotiana benthamiana* plants. Total proteins were extracted 3 days post-infiltration using lysis buffer (50 mM Tris-HCl, pH 7.5, 150 mM NaCl, 5 mM EDTA, 0.1% Triton X-100, 0.2% NP-40, 1 mM PMSF, and 1× protease inhibitor cocktail).

Protein extracts were incubated with GFP-nanobody magnetic beads (LabLead, Shanghai, China) at 4 °C for 3 h with gentle rotation. After five washes with lysis buffer, immunoprecipitated proteins were eluted with 2× SDS loading buffer, separated by SDS–PAGE, and transferred onto PVDF membranes. Western blotting was performed using anti-HA (1:5000) and anti-GFP (1:5000) antibodies, followed by HRP-conjugated secondary antibodies (Thermo Fisher Scientific, Waltham, MA, USA, 1:10,000). Signals were detected using an ECL chemiluminescence kit (Thermo Fisher Scientific, Waltham, MA, USA). All original Western blot images for the Co-IP assay are provided in Supplementary Materials (Figure S1).

### 2.5. Protein Purification and Pull-Down Assay

The full-length coding sequence of PGD2 was subcloned into the pET28a vector to generate pET28a-PGD2 for prokaryotic expression. The full-length ORFs of G6PD6 and GFP were inserted into the pMAL-c5x vector to produce MBP-G6PD6 and MBP-GFP, respectively. All constructs were transformed into *Escherichia coli* strain BL21 (DE3).

For PGD2-HIS expression, BL21 cells harboring pET28a-PGD2 were induced with 0.5 mM IPTG at 15 °C for 24 h, lysed by sonication, and purified using His60 Ni Superflow resin (Takara, Kyoto, Japan). BL21 cells expressing MBP-G6PD6 or MBP-GFP were induced with 1 mM IPTG at 37 °C for 4 h and purified using amylose resin (NEB, Ipswich, MA, USA).

For the pull-down assay, purified PGD2-HIS proteins were first immobilized on Mag-Beads His-Tag resin (Sangon, Shanghai, China) by incubation at 4 °C for 1 h. The beads were then incubated with MBP-G6PD6 or MBP-GFP (negative control) at 4 °C for 3 h. After three washes, the bound proteins were eluted in 2× SDS loading buffer, separated by SDS-PAGE, transferred onto PVDF membranes (Millipore, Billerica, MA, USA), and detected using appropriate antibodies. Uncropped original images of Western blot analyses for the pull-down assay are included in Supplementary Materials (Figure S2).

### 2.6. Interaction Complex Modeling

The three-dimensional structures of PGD2 and G6PD6 were predicted using AlphaFold2 v2.3.2 (DeepMind, London, UK) [17]. Protein–protein interaction models were generated using AlphaFold2-Multimer v3.0 (DeepMind, London, UK) [18] and AF2Complex v1.4.1 [19]. These tools were employed to predict the PGD2–G6PD6 heterodimeric interface and evaluate the structural feasibility of the interaction.

## 3. Results

### 3.1. Overexpression of PGD2 Rescues the Defective Phenotype of adh2 Mutants

To explore the mechanisms underlying aromatic amino acid (AAA) biosynthesis, we previously performed Ethyl Methanesulfonate (EMS) mutagenesis screening using the *adh2* mutant, which exhibits a clear growth defect but retains normal fertility [12]. This screen identified a suppressor mutant carrying a point mutation in PGD2. Our earlier work demonstrated that this mutation enhanced catalytic turnover by facilitating product release, thereby increasing metabolic flux toward downstream AAA biosynthesis [14]. These findings led us to propose that PGD2 may function within a protein complex to coordinate AAA synthesis.

**Figure 1:**
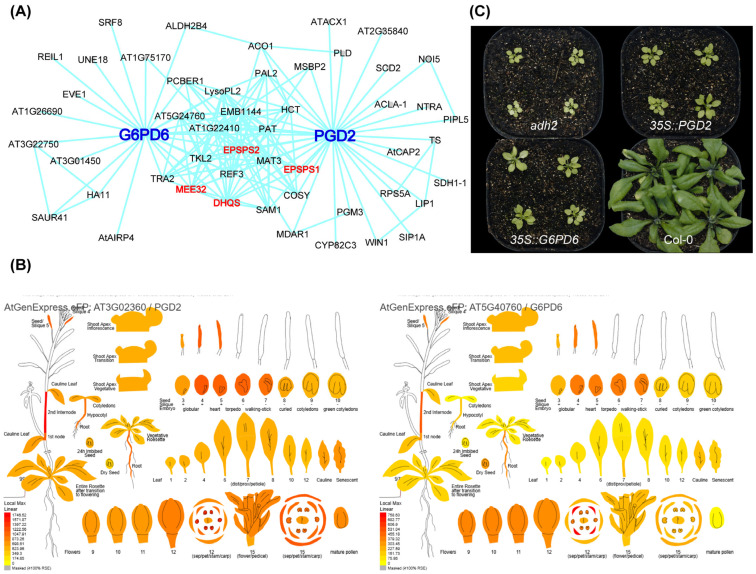
Co-expression patterns of *PGD2* and *G6PD6* and phenotypic rescue in the *adh2* mutant. (**A**) Gene co-expression network showing *PGD2* and *G6PD6* clustered within the same shikimate- and lignin-related module. (**B**) Tissue-specific expression profiles of *PGD2* (**left**) and *G6PD6* (**right**) across Arabidopsis organs and developmental stages, showing synchronized expression patterns. Expression data were retrieved from the TAIR eFP Atlas (https://www.bar.utoronto.ca/efp//cgi-bin/efpWeb.cgi, accessed on 9 October 2024). (**C**) Overexpression of *PGD2* or *G6PD6* partially rescues the defective phenotype of the aromatic amino acid-deficient mutant *adh2*. Col-0 and *adh2* plants serve as controls.

Analysis of the AtGGM2014 *Arabidopsis* gene co-expression network revealed that PGD2 and G6PD6 are co-enriched within a module associated with shikimate and lignin biosynthetic genes, displaying a strong co-expression relationship (Figure 1A). Consistently, expression patterns extracted from the TAIR eFP atlas [20] showed that PGD2 and G6PD6 exhibit highly similar spatiotemporal expression profiles, with high abundance in photosynthetic tissues and low abundance in roots (Figure 1B). This coordinated expression pattern—consistent with the chloroplast-localized synthesis of AAAs—suggests a functional relationship rather than a coincidental correlation.

Further supporting this idea, overexpression of G6PD6 in the *adh2* background also partially rescued the mutant phenotype (Figure 1C). Together, these data led us to hypothesize that PGD2 and G6PD6 may directly interact to jointly regulate AAA production.

### 3.2. PGD2 Physically Interacts with G6PD6 in the Cytoplasm via Its C-Terminal Region

**Figure 2:**
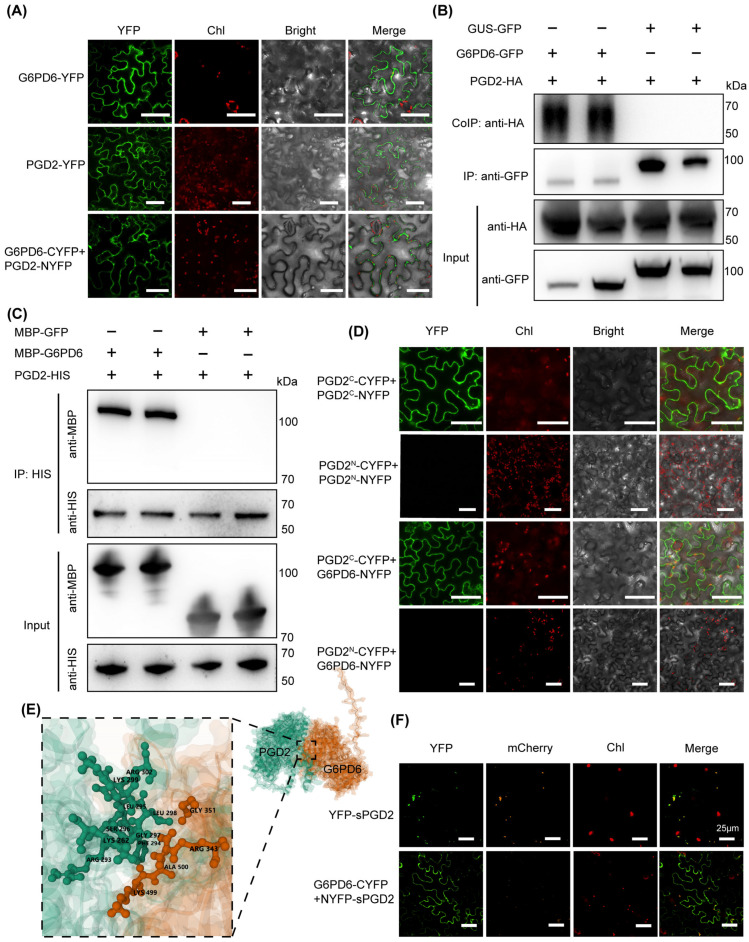
Interaction Validation of PGD2 and G6PD6. (**A**) Subcellular localization and BiFC assays indicate PGD2 and G6PD6 interact in the cytoplasm. Bar = 50 μm. (**B**) Co-IP assay confirms their interaction in vivo. (**C**) Pull-down assay shows their direct binding in vitro. (**D**) BiFC and domain analysis of PGD2-G6PD6 interaction. Bar = 50 μm. (**E**) Predicted structural model of the PGD2-G6PD6 complex. (**F**) Truncated PGD2 interacts with G6PD6 in the cytoplasm. Representative images under YFP, chlorophyll (Chl), and mCherry (the peroxisome marker PGL3) channels and the merged signals are shown. Bar = 50 μm.

To test this hypothesis, we first examined the subcellular localization of the two proteins. Full-length PGD2 and G6PD6 fused to C-terminal YFP were both localized to the cytoplasm (Figure 2A), suggesting that their functional interaction likely occurs in this compartment. A BiFC assay performed in *Nicotiana benthamiana* leaves supported this inference: co-expression of G6PD6-CYFP and PGD2-NYFP generated a clear YFP fluorescence signal in the cytoplasm, directly demonstrating their interaction in vivo (Figure 2A).

This interaction was further validated by both in vivo and in vitro approaches. Co-immunoprecipitation showed that GFP-tagged G6PD6 successfully pulled down HA-tagged PGD2 from tobacco extracts (Figure 2B), indicating that the two proteins form a complex in plant cells. Consistently, purified HIS-PGD2 protein specifically bound immobilized MBP-G6PD6 in pull-down assays (Figure 2C), confirming a direct physical interaction.

To identify the interaction region, we performed domain mapping of PGD2. Only the C-terminal region of PGD2 interacted with G6PD6 (Figure 2D), and this same region also mediated PGD2 homodimerization. Structural modeling further predicted that PGD2 residues PHE^294^, GLY^297^, and LEU^298^ interact with G6PD6 residues GLY^351^, LYS499, and ALA^500^, forming a stable interface (Figure 2E). This structural model provides corroboration for the interaction validated by BiFC, Co-IP, and pull-down assays, while laying a foundation for subsequent functional validation of key interface residues and screening of potential additional complex components.

Previous studies reported that PGD2 shows dual localization in both the cytoplasm and peroxisomes [10,11]. Consistent with this, truncated PGD2 lacking the first 15 N-terminal amino acids co-localized with a peroxisomal marker [21]. However, interaction assays demonstrated that this truncated PGD2 still interacted with G6PD6 exclusively in the cytoplasm, indicating that the cytoplasm is the primary functional compartment for PGD2–G6PD6 association.

## 4. Discussion

Aromatic amino acids (AAAs) serve not only as essential building blocks for protein synthesis but also as precursors for diverse secondary metabolites, including flavonoids, lignin, and auxin [13]. Thus, plants must precisely coordinate AAA biosynthesis with developmental and environmental cues [22]. The shikimate pathway is central to AAA production and relies heavily on metabolic input from the oxidative pentose phosphate (OPP) pathway, particularly the supply of erythrose-4-phosphate (E4P) and reducing power in the form of NADPH [7]. Although the metabolic relationship between these two pathways has long been recognized, the molecular basis for their coordinated regulation has remained unclear.

In this study, we identified a previously unknown physical and functional connection between two sequential OPP pathway enzymes—G6PD6, the rate-limiting enzyme initiating carbon entry into the pathway [23], and PGD2, which catalyzes the subsequent oxidative decarboxylation step [10]. We showed that PGD2 interacts directly with G6PD6 in the cytoplasm through its C-terminal region, forming a functional protein complex that enhances AAA biosynthesis. This finding provides direct evidence supporting the long-standing hypothesis that plants employ multienzyme assemblies to channel metabolic intermediates and ensure efficient flux through essential biosynthetic pathways.

Our findings also integrate well with genetic and expression evidence. PGD2 and G6PD6 display highly synchronized spatiotemporal expression patterns, and overexpression of either gene partially rescues the growth defects of the *adh2* mutant, which is impaired in AAA biosynthesis. These observations suggest that the PGD2–G6PD6 complex acts as a key metabolic node linking central carbon metabolism with the shikimate pathway. While previous studies have characterized the enzymatic activities of G6PD6, PGD2, and shikimate pathway components individually, the present work revealed a unifying protein-level mechanism that connects these processes into a coordinated metabolic module.

Future research should explore whether additional enzymes participate in this complex, how its assembly is regulated, and whether it interfaces directly with shikimate pathway enzymes to form higher-order metabolic scaffolds. Such studies will deepen our understanding of how plants integrate redox balance, metabolic flux, and biosynthetic demand. In addition, elucidating the regulatory logic of this complex may provide promising strategies for enhancing AAA-derived metabolites and improving crop nutritional and industrial traits.

## 5. Conclusions

In this study, we reveal a direct physical and functional interaction between two sequential OPP enzymes, PGD2 and G6PD6. The proteins form a cytoplasmic complex via C-terminal region of PGD2, and overexpression of either gene partially rescued the *adh2* mutant phenotype. This interaction enhances OPP-derived inputs, including erythrose-4-phosphate and NADPH, supporting aromatic amino acid biosynthesis and maintaining redox balance. Our findings uncover a mechanism linking central carbon metabolism with the shikimate pathway and illustrate how enzyme associations optimize AAA production.

## Data Availability

All of the relevant data used in this study are included in this manuscript. The corresponding authors can be contacted if any further information is needed.

## Supplementary Materials

The following supporting information can be downloaded at https://www.mdpi.com/article/10.3390/biology14121712/s1. Figure S1: All original Western blot images for the Co-IP assay; Figure S2: Uncropped original images of Western blot analyses for the pull-down assay.
