# Supplementary material for: The Interaction Between PGD2 and G6PD6 Is Involved in Aromatic Amino Acid Synthesis
Source: Biology (Basel). 2025 Nov 30;14(12):1712. doi: 10.3390/biology14121712 (PMC12730105; doi:10.3390/biology14121712)
Supplement: Supplementary file 1 [file biology-14-01712-s001.zip › biology-4010611-supplementary.pdf]

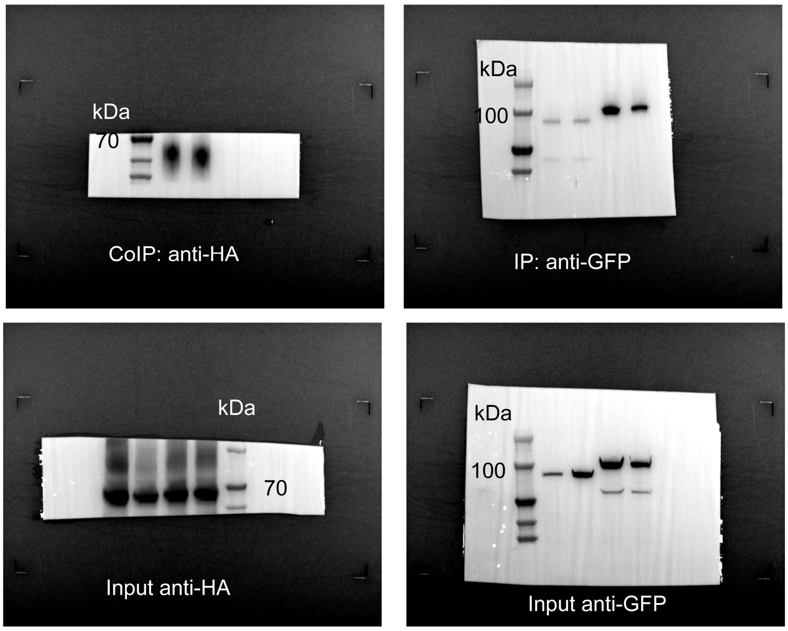

Figure S1. All original Western blot images for the Co-IP assay.

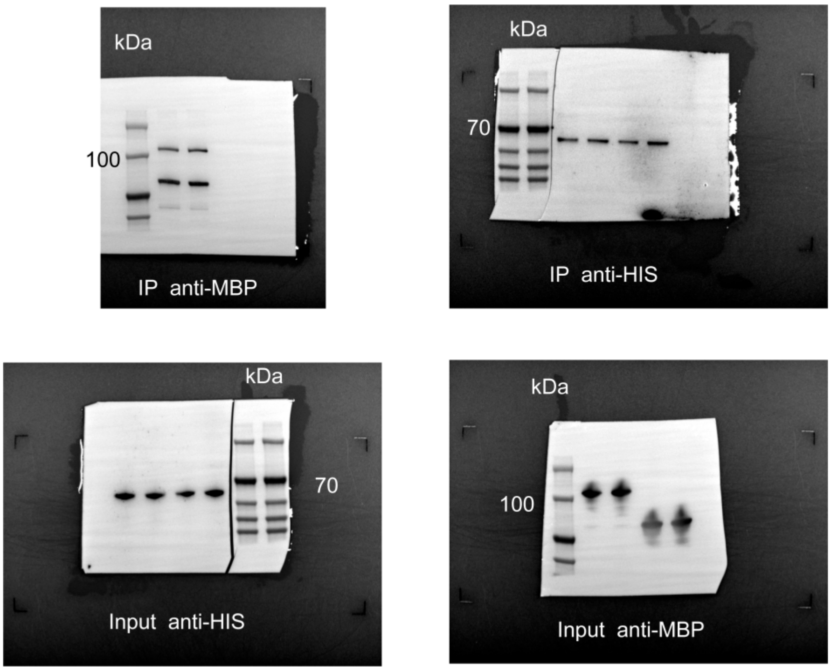

Figure S2. Uncropped original images of Western blot analyses for the pull-down assay.
